# Cognitive–behavioral therapy for management of mental health and stress-related disorders: Recent advances in techniques and technologies

**DOI:** 10.1186/s13030-021-00219-w

**Published:** 2021-10-03

**Authors:** Mutsuhiro Nakao, Kentaro Shirotsuki, Nagisa Sugaya

**Affiliations:** 1 Department of Psychosomatic Medicine, School of Medicine, International University of Health and Welfare, 4-3, Kozunomori, Narita-shi, Chiba, 286-8686 Japan; 2 Graduate School of Human and Social Sciences, Musashino University, Tokyo, Japan; 3 Unit of Public Health and Preventive Medicine, School of Medicine, Yokohama City University, Yokohama, Japan

**Keywords:** Biopsychosocial approach, Cognitive–behavioral therapy, Stress management

## Abstract

Cognitive–behavioral therapy (CBT) helps individuals to eliminate avoidant and safety-seeking behaviors that prevent self-correction of faulty beliefs, thereby facilitating stress management to reduce stress-related disorders and enhance mental health. The present review evaluated the effectiveness of CBT in stressful conditions among clinical and general populations, and identified recent advances in CBT-related techniques. A search of the literature for studies conducted during 1987–2021 identified 345 articles relating to biopsychosocial medicine; 154 (45%) were review articles, including 14 systemic reviews, and 53 (15%) were clinical trials including 45 randomized controlled trials. The results of several randomized controlled trials indicated that CBT was effective for a variety of mental problems (e.g., anxiety disorder, attention deficit hypersensitivity disorder, bulimia nervosa, depression, hypochondriasis), physical conditions (e.g., chronic fatigue syndrome, fibromyalgia, irritable bowel syndrome, breast cancer), and behavioral problems (e.g., antisocial behaviors, drug abuse, gambling, overweight, smoking), at least in the short term; more follow-up observations are needed to assess the long-term effects of CBT. Mental and physical problems can likely be managed effectively with online CBT or self-help CBT using a mobile app, but these should be applied with care, considering their cost-effectiveness and applicability to a given population.

## History of cognitive–behavioral therapy (CBT)

CBT is a type of psychotherapeutic treatment that helps people to identify and change destructive or disturbing thought patterns that have a negative influence on their behavior and emotions [1]. Under stressful conditions, some individuals tend to feel pessimistic and unable to solve problems. CBT promotes more balanced thinking to improve the ability to cope with stress. The origins of CBT can be traced to the application of learning theory principles, such as classical and operant conditioning, to clinical problems. So-called “first-wave” behavioral therapy was developed in the 1950s [2]. In the US, Albert Ellis founded rational emotive therapy to help clients modify their irrational thoughts when encountering problematic events, and Aaron Beck employed cognitive therapy for depressed clients using Ellison’s model [3]. Behavioral therapy and cognitive therapy were later integrated in terms of theory and practice, leading to the emergence of “second-wave” CBT in the 1960s. The first- and second-wave forms of CBT arose via attempts to develop well-specified and rigorous techniques based on empirically validated basic principles [4]. From the 1960s onward, the dominant psychotherapies worldwide have been second-wave forms of CBT. Recently, however, a third-wave form of CBT has attracted increasing attention, leading to new treatment approaches such as acceptance and commitment therapy, dialectical behavior therapy, mindfulness-based cognitive therapy, functional analytic psychotherapy, and extended behavioral activation; other forms may also exist, although this is subject to conjecture [4]. In a field of psychosomatic medicine, it has been reported that cognitive restructuring is effective in improving psychosomatic symptoms [5], exposure therapy is suitable for a variety of anxious disease conditions like panic disorder and agoraphobia [6], and mindfulness reduces stress-related pain in fibromyalgia [7]. Several online and personal computer-based CBT programs have also been developed, with or without the support of clinicians; these can also be accessed by tablets or smartphones [8]. Against this background, this review focused on the effectiveness of CBT with a biopsychosocial approach, and proposed strategies to promote CBT application to both patient and non-patient populations.

## Research on CBT

**Fig. 1:**
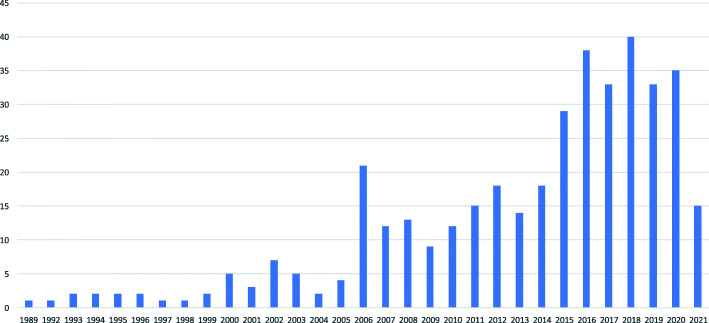
Number of articles per year identified by a PubMed search from 1989 to the present

Using “CBT “and “biopsychosocial” as PubMed search terms, 345 studies published between January 1987 and May 2021 were identified (Fig. 1); 14 of 154 review articles were systemic reviews, and 45 of 53 clinical trials were randomized controlled trials. Most clinical trials recruited the samples from patient populations in order to assess specific diseases, but some targeted at those from non-patient populations like a working population in order to assessing mind-body conditions relating to sick leave [9]. The use of biopsychosocial approaches to treat chronic pain is shown to be clinically and economically efficacious [10]; for example, CBT is effective for chronic low-back pain [11]. The prevalence of chronic low-back pain, defined as pain lasting for more than 3 months, was reported to be 9% in primary-care settings and 7–29% in community settings [12]. Chronic low-back pain is not only prevalent, but is a source of significant physical disability, role impairment, and diminished psychological well-being and quality of life [11]. Interestingly, according to the results of our own study [13], CBT was effective among hypochondriacal patients without chronic low-back pain, but not in hypochondriacal patients with chronic low-back pain. These group differences did not seem to be due to differences in the baseline levels of hypochondriasis. Although evidence has suggested that both hypochondriasis and chronic low-back pain can be treated effectively with CBT [10, 11, 14], this has not yet been validated. Chronic low-back pain may be associated with a variety of conditions, including anxiety, depression, and somatic disorders such as illness conviction, disease phobia, and bodily preoccupation. The core psychopathology of hypochondriacal chronic low-back pain should be clarified to promote adequate symptom management [13].

Since 2000, Cochrane reviews have evaluated the effectiveness of CBT for a variety of mental, physical, and behavioral problems. Through a search of the Cochrane Library database up to May 2021 [15], 124 disease conditions were assessed to clarify the effects of CBT in randomized controlled trials; the major conditions for which CBT showed efficacy are listed in Table 1. These include a broad range of medical problems such as psychosomatic illnesses (e.g., chronic fatigue syndrome, irritable bowel syndrome, and fibromyalgia), psychiatric disorders (e.g., anxiety, depression, and developmental disability), and socio-behavioral problems (drug abuse, smoking, and problem gambling). For most of these conditions, CBT proved effective in the short term after completion of the randomized controlled trial. Although the number of literature was still limited, some studies have reported significant and long-term treatment effects of CBT on some aspects of mental health like obsessive-compulsive disorder [16] 1 year after the completion of intervention. Future research should investigate the duration of CBT’s effects and ascertain the optimal treatment intensity, including the number of sessions.

**Table 1:** Example diseases and problems for which CBT is expected to be effective (Cochrane reviews)

Major disease conditions	Summary of evidence	Update
Psychiatric disorders: Depression, general	‘Third -wave’ CBT as effective treatment of acute depression Reduced depressive symptoms in dementia and mild cognitive impairment Improved response and remission rates for treatment-resistant depression Reduced depressive symptoms in children with long-term physical conditions Reduced depressive symptoms in chronic obstructive pulmonary disease Reduced depressive symptoms in dialysis patients Reduced the number of sickness absence days in workers	October 2013 January 2014 May 2018 December 2018 March 2019 December 2019 October 2020
Anxiety, general Obsessive–compulsive disorder Panic disorder	Reduced anxiety symptoms in adults by “media-delivered CBT” (self-help) Reduced anxiety symptoms in dementia and mild cognitive impairment Reduced anxiety symptoms in adults by therapist-supported internet CBT Reduced anxiety symptoms in children with long-term physical conditions Effective for attention control in children and adolescents Effective in children and adolescents with this disorder Effective in adults with this disorder Efficacy of both CBT alone and CBT and antidepressants Efficacy of both CBT and benzodiazepines	September 2013 January 2014 March 2016 December 2018 November 2020 October 2006 April 2007 January 2007 January 2009
Post-traumatic stress disorder (PTSD) Social anxiety disorder	Effective in children and adolescents for up to 1 month following CBT Reduced clinician-assessed PTSD symptoms in adults Reduced PTSD symptoms when used as couple and family therapies Reduced social phobia via brief CBT	December 2012 December 2013 December 2019 September 2018
Acute stress disorder	Reduced acute traumatic stress symptoms via brief trauma-focused CBT	March 2010
Attention deficit–hyperactivity disorder	Beneficial for treating adults with this disorder in the short term	March 2018
Bulimia nervosa Hypochondriasis Somatoform disorder	Efficacy of a specific manual-based form of CBT for bulimia nervosa Reduced hypochondriacal symptoms and general functioning Reduced symptom severity in adults with somatoform disorders	October 2009 October 2007 November 2014
Physical diseases: Breast cancer	Improved survival at 12 months (metastatic) Favorable effects on anxiety, depression and mood disturbance (non-metastatic)	June 2013 May 2015
Chronic fatigue syndrome Fibromyalgia	Reduced fatigue symptoms Reduced pain, negative mood, and disability	July 2008 September 2013
Irritable bowel syndrome Recurrent abdominal pain	Reduced symptoms of irritable bowel syndrome and improved quality of life Reduced pain in the short term in children and adolescents	January 2009 January 2017
Tinnitus	Reduced negative impacts on quality of life and depression	January 2020
Behavioral and other problems: Antisocial behaviors Benzodiazepine use Burden of care for dementia Early behavioral problems Needle-related problems Obesity and overweight Occupational stress Problem gambling Self-harm Sexual abuse	Reduced antisocial behaviors in young people in the short term Effective in the short term for reducing benzodiazepine harmful use Reduced psychological stress in family caregivers of people with dementia Improved child conduct problems, parental mental health, and parenting skills Reduced children’s needle-related pain and distress in children and adolescents Reduced weight, predominantly useful when combined with diet and exercise Reduced stress at work in healthcare workers Reduced pathological and problem gambling behaviors immediately after CBT Resulted in fewer adults repeatedly self-poisoning and self-injuring Reduced symptoms of PTSD, anxiety, and depression in children	October 2007 May 2015 November 2011 February 2012 October 2018 April 2005 April 2015 November 2012 May 2016 May 2012
Smoking	Effective for smoking cessation in indigenous populations	January 2012

## Future directions for CBT application in biopsychosocial domains

In Japan, CBT for mood disorders was first covered under the National Health Insurance (NHI) in 2010, and CBT for the following psychiatric disorders was subsequently added to the NHI scheme: obsessive–compulsive disorder, social anxiety disorder, panic disorder, post-traumatic stress disorder, and bulimia nervosa [17]. The treatment outcomes and health insurance costs for these six disorders should be analyzed as the first step, for appropriate allocation of medical resources according to disease severity and complexity [18]. In Japan, health insurance coverage is provided only when physicians apply for remuneration. A system promoting nurse involvement in CBT delivery [19], as well as shared responsibility between the CBT instructor and certified psychologists (or even a complete shift from physicians to psychologists), has yet to be established. Information and communication technology (ICT) devices may allow CBT delivery to be shared between medical staff and psychologists, in medical, community and self-help settings [8]. The journal BioPsychoSocial Medicine published 334 relevant articles up to the end of May 2021, 112 (33.5%) of which specifically addressed CBT [20]. CBT is a hot topic in biopsychosocial medicine, and more research is required to encourage its application to clinical and general populations.

## Data Availability

Not applicable.

## Abbreviations

CBT: Cognitive–behavioral therapy
ICT: Information and communication technology
NHI: National Health Insurance
PTSD: Post-traumatic stress disorder
